# Demonstrations vs. Lectures

**Published:** 1871-06

**Authors:** C. M. Wright


### DEMONSTRATIONS *vs.* LECTURES.

BY C. M. WRIGHT, D. D. S.

Read before the Mad River Dental Society, May 9, 1871.

I do not know that I can put down in readable form, the thoughts that have occupied me for some time on a subject connected with dental education. As I can not be present at this meeting of the Mad River Valley Dental Society in person, I will make the effort to send a thought as a proxy. I want to say a word in favor of demonstrations, of clinics, of the work of the hands, of visible methods of giving instruction in a practical and mechanical or artistic art, and should like to show the superiority of this method of instruction over the old system of lectures pursued in most of our colleges and universities, and especially in our professional schools. The lecture system has been so long pursued, that veneration for an established custom may prejudice our minds in its favor without much thought; yet while it has some advantages, and while thousands of learned men have used this method of gaining and of giving instruction, it seems to me that too much stress is laid upon the importance of this custom, and too little attention is given to the more important, and more successful, and more rational method of giving instruction in practical studies. If we desire to teach metaphysics, or a system of religion or mental philosophy; if we want to teach history of men, science or art, the lecture system as pursued in the German universities, is probably a good way. It brings the student into direct contact with the living thoughts of a teacher, knowledge and renown; it helps, perhaps, better than reading, to make thought burn and tingle in his mind; it stimulates the student and fixes his attention; it calls up in his own mind connective thoughts, and helps him think. This will apply, of course, to lectures on any subject, theoretic or practical—but suppose the subject is one that deals only with an art—

a manual art. Suppose we wish to teach our student surgery—is it the best way? A man might listen attentively to lectures through twenty courses, on surgery, and yet faint at the sight of the knife and the first drop of blood. He might be versed in the nomenclature of anatomy, and yet have no knowledge of the track of a single vein or artery. Knowledge of an art is not a mass of principles stored in the memory. Look at the crowds of young men that leave our medical colleges every winter with diplomas in their hands. Would you trust one of them with the life of a favorite child? He may know more names of diseases, more histories of symptoms, more opinions of medical writers than your old family physician. He, (the young graduate) has come home, crammed with medical lore, and filled fresh from the fount with lectures—but the old doctor knows the tongue, the eye, the pulse. He *knows* quinia and blue mass. His knowledge is a knowledge of facts—of experience. He has forgotten his lectures entirely, but he remembers his *cases*. In dentistry, how should we teach? I will not speak of anatomy, physiology, etc., but only of mechanical dentistry. Mechanical dentistry is an art. The *science* is comprised in the *art*. How can we best teach this art? By lectures? The answer to these questions, it seems to me is plain, if we think for a minute what we want our students to know, and what they want to know when they are standing by the side of their first patient. Shall one of our students, with a diploma from a dental college, stand by his patient, and remember what Professor A or B said about filling approximal cavities, or about extracting, and inserting a partial denture, and with untrained hands and eyes, and muscles and judgment, attempt to put into practice his memories? By no means. His efforts would make any of us blush for our colleges. Then why lecture him for five months, quiz his memory, and give him a diploma? I can not put this subject in the strong light that I would like to. I can not express it as decidedly as I feel about it. It would not be polite. But it seems to me that the lecture system in all practical or exact arts or sciences is carried altogether too far. If I want to learn geology, I don't want to hear Professor Rock talking about strata, substrata, or anything of the sort. I want to follow him out into the fields, and with hammer and blasting powder, work and see, and think and feel geology. If I want to learn botany, or astronomy, or painting, or architecture, I want to *handle* and *see*, that I may know these things. Is this not true in mechanical and operative dentistry? In books and lectures we probably get the best thoughts of those who make books or deliver lectures—but if we want to teach farming, or navigation, or house building, or picture painting, we must take our students with us, and make them put their hands to the plow and the wheel, the saw and the brush, and demonstrate our principles in our practice.

Dentistry should be taught in the same way. If we must have lectures in our dental colleges to keep up the custom, and to sustain a false notion of the dignity of a college, let them be few, and the demonstrations many. How often has it been illustrated, that a five minutes talk of explanation and demonstration by a sick bed, has given the student more and better ideas than a months' close attention to lectures. How often is it true that at our dental associations, a five minutes peep at a great operator in a clinic, will teach us more of filling teeth than all our books and all our talks? If what I have written is true, when you send your student to a dental college, select the one that teaches practical dentistry in a practical way, and if your own conscience don't applaud you, your student in after days will.

